# Optimization for Liquid-Liquid Extraction of Cd(II) over Cu(II) Ions from Aqueous Solutions Using Ionic Liquid Aliquat 336 with Tributyl Phosphate

**DOI:** 10.3390/ijms21186860

**Published:** 2020-09-18

**Authors:** Lai Yee Lee, Norhashimah Morad, Norli Ismail, Amir Talebi, Mohd Rafatullah

**Affiliations:** School of Industrial Technology, Universiti Sains Malaysia, Gelugor 11800, Penang, Malaysia; lly15_tec003@student.usm.my (L.Y.L.); nhashima@usm.my (N.M.); norlii@usm.my (N.I.); amirtalebi@usm.my (A.T.)

**Keywords:** heavy metal separation, ionic liquid, extraction, masking agent, liquid-liquid extraction, selective extraction

## Abstract

This study investigates the separation of two heavy metals, Cd(II) and Cu(II), from the mixed synthetic feed using a liquid-liquid extraction. The current study uses tri-octyl methylammonium chloride (Aliquat 336) as the extractant (with tributyl phosphate (TBP) as a phase modifier), diluted in toluene, in order to investigate the selective extraction of Cd(II) over Cu(II) ions. We investigate the use of ethylenediaminetetraacetic acid (EDTA) as a masking agent for Cu(II), when added in aqueous feed, for the selective extraction of Cd(II). Five factors that influence the selective extraction of Cd(II) over Cu(II) (the equilibrium pH (pH_eq_), Aliquat 336 concentration (Aliquat 336), TBP concentration (TBP), EDTA concentration (EDTA), and organic to aqueous ratio (O:A)) were analyzed. Results from a 2^5–1^ fractional factorial design show that Aliquat 336 significantly influenced Cd(II) extraction, whereas EDTA was statistically significant for the antagonistic effect on the E% of Cu(II) in the same system. Moreover, results from optimization experiment showed that the optimum conditions are Aliquat 336 concentration of 99.64 mM and EDTA concentration of 48.86 mM—where 95.89% of Cd(II) was extracted with the least extracted Cu(II) of 0.59%. A second-order model was fitted for optimization of Cd(II) extraction with a R^2^ value of 0.998, and ANOVA results revealed that the model adequately fitted the data at a 5% significance level. Interaction between Aliquat 336 and Cd(II) has been proven via FTIR qualitative analysis, whereas the addition of TBP does not affect the extraction mechanism.

## 1. Introduction

Stringent environmental regulations and depletion of the world’s mineral resources have urged for the removal and recovery of heavy metals from the metallurgical production and hydrometallurgical processing waste and secondary sources in complex leach solutions. Despite their toxicity, Cd and Cu are used in industries, such as metal refining, mining, electroplating, and manufacturing of alloys. Cd is often found in industrial waste by-products, such as Cd-rich dust, Cu-Cd slag, and hydrometallurgical leachates, along with other heavy metals, e.g., Cu, Ni, Zn, etc. [1]. Selective separation and recovery of Cd from wastewater containing various metallic constituents can be achieved by chemical precipitation, adsorption, ion exchange, solvent extraction, electrolysis, etc. [2]. Different metal species with almost identical valence configurations in the same mixture allows co-transport, and it makes selective extraction a tough challenge. Selective extraction of Cd(II) is feasible in the presence of Zn(II), Ni(II), Co(II), Mn(II), Fe(II), Ca(II), and Mg(II), but Cu(II) and Pb(II) were found to be co-extracted with Cd(II) [3]. The presence of Cd in the same mixture was found to interrupt the physiological balance of other metals [4].

Liquid-liquid extraction (LLE) is one of the most versatile techniques used for the selective separation, recovery, and purification of aqueous media containing metal ions [5]. LLE is a simple and quick method with low operational cost [6]. LLE utilizes the principle of analyte (metal cation) distribution ratios between two immiscible liquids (generally consist of one organic and one aqueous phase) in contact with each other to achieve separation. The separation of metal within a multi-element mixture is known as selectivity. To justify the efficiency of LLE in selective separation of one metal over the other metal, the distribution ratios and separation factors of metals using selected extractant are used by most researchers [7,8]. Several studies have been reported in the literature on different combinations of organic extractants have been intensively investigated for the extraction of Cd(II) from synthetic solutions, industrial wastewaters, and complex leach solutions. Organophosphorus-based extractants, such as di-(2-ethylhexyl) phosphoric acid (D2EHPA), 2-ethylhexyl phosphonic acid mono-2-ethylhexyl ester (PC88A), and di-2,4,4-trimethylpentyl phosphinic acid (Cyanex 272), were used to extract of Cd(II) from synthetic sulfate solutions by cation exchange mechanism [9]. Free fatty acid-rich oil derived from palm kernel distillate had been introduced for Cu(II) extraction, as green and renewable extractants without the need of diluent for its low melting point, low density, low water solubility, and moderate viscosity [10]. However, the new fatty acid has not been tested with the separation of other metals, and its selectivity for other metals is not specified. Ionic liquids (IL) are known as task-specific extractants and have been highlighted in various scientific publications for their improved and adjustable physicochemical properties, such as thermal stability, high polarity, negligible vapor pressure, non-flammability, and wide range of miscibility with other organic solvents [11,12]. Quaternary ammonium-based IL, such as tri-octyl methylammonium chloride (Aliquat 336), and phosphonium-based IL, e.g., trihexyl(tetradecyl)phosphonium chloride (Cyphos IL101), were used to extract Cd(II) [13]. Rapid extraction equilibria were achieved, where 99% of Cd(II) ions were extracted using 0.2 M Aliquat 336 and 0.04 M Cyphos IL101 in kerosene, respectively. Trihexyl(tetradecyl)phosphonium bis(2,4,4-trimethylpentyl) phosphinate (Cyphos IL 104) was used for separation of Cd(II) over Cu(II), Co(II) and Ni(II) and the separation factors were found in order of S_Cd/Cu_ < S_Cd/Co_ < S_Cd/Ni_ [14].

Neutral extracting agents like tributyl phosphate (TBP) and trioctylphosphine oxide (TOPO) have high extraction coefficients for many metals and organic solutions, but low selectivity when used as sole extractant. TBP has been discovered to control third-phase formation, as a phase modifier. D2EHPA-TBP has been proven for the improved extraction of several metal cations, such as Cu(II) [15,16], Co(II) [17,18], Fe(III) [19], Ni(II) [20], Zn(II) [21]. There is no literature traceable regarding the extraction of Cd(II) using the combination of Aliquat 336 and TBP. Application of masking agent enhances the selective separation of metal ions by means to suppress the interference of unwanted constituents in a system without forming elaborate separation. Masking agents are also metal-complexing agents, introduced to improve the separation factor in the extraction procedures. Separation factor of Cd(II) over Zn(II) was increased by more than 500 times using D2EHPA as extractant and an aqueous hexadentate ligand with nitrogen donors, *N*,*N*,*N*′,*N*′-tetrakis(2-pyridylmethyl)ethylene diamine (TPEN) as a masking agent for Zn(II) [22]. Ethylenediaminetetraacetic acid (EDTA) has been proved as the most useful masking agent, by forming anionic complexes with several metal ions. EDTA acted as a masking agent in the source phase for Fe(III), Cu(II), Ni(II), and Zn(II) during the selective permeation of uranium using sodium carbonate as the receiving phase [23]. 0.1 M EDTA was added into ammonia buffer at the stripping phase at a ratio of 4 to 1 to separate Cd(II) from Zn(II) and Ni(II) [13].

The investigation of the capability of Aliquat 336-TBP in extracting Cd(II) and the efficiency of EDTA in suppressing the co-extraction of Cu(II) has not been reported in the literature. The current work aims to find out the selective extraction of Cd(II) over Cu(II) ions by using Aliquat 336 (extractant) with TBP (phase modifier) and EDTA as a masking agent. Various process variables affecting the extraction were studied and optimized using response surface methodology (RSM). RSM has been proven to be an effective statistical tool to evaluate the interaction between variables [24,25]. In this study, the two-level fractional factorial design was used for screening experiments, whereas central composite design was used for the optimization of significant parameters. Analysis of the second-order model was conducted to achieve the optimum response. Qualitative Fourier transform infrared (FTIR) measurement was recorded on the extractant and their combination to compare the change of bonds in the organic phase after extraction.

## 2. Materials and Methods

### 2.1. Chemicals and Reagents

Copper sulfate pentahydrate (CuSO_4_·5H_2_O) (≥99.6% purity) and cadmium sulfate hydrate (CdSO_4_·H_2_O) (≥98% purity) were obtained from Merck. The organic extractants used to extract the metal ion were TBP (≥99% purity), and Aliquat 336 (≥95% purity) from Sigma-Aldrich. The dilution of organic extractant was performed using commercial toluene purchased from Sigma-Aldrich.

Nitric acid (HNO_3_) (≥65%), sulfuric acid (H_2_SO_4_) (≥98%), ethylenediaminetetraacetic acid (EDTA) (≥98%), sodium hydroxide (NaOH) (≥99%) and sodium sulfate (Na_2_SO_4_) (≥99%) were purchased from Merck. Glassware was cleansed with Decon 90 and washed before soaking in 5% HNO_3_. Deionized water was used for final washing of glassware and to prepare all the aqueous mixtures.

### 2.2. Equipment

Aqueous and organic phases were mixed using a digital overhead stirrer (IKA, Microstar 7.5 control). Initial and final equilibrium pH (pH_eq_) readings of aqueous phase were measured by pH meter (Sension+ pH3, Hach, Loveland CO, USA). The Cd(II) and Cu(II) ions concentration before and after extraction studies were analyzed separately using a flame atomic absorption spectrophotometer (FAAS) (AA-7000, Shimadzu, Tokyo, Japan) after appropriate filtration and dilution. Air-acetylene (Air-C_2_H_2_) flame of 2300 °C was used for atomization of all samples. Analyses of Cd(II) and Cu(II) were conducted, based on Table 1.

FTIR spectrometer (Frontier™, Perkin Elmer, MA, USA) with universal attenuated total reflectance polarization (FTIR-ATR) was operated in the mid-infrared region with wavenumbers spanning from about 600 cm^−1^ to 4000 cm^−1^ for investigation of infrared spectra of the organic phase before and after extraction.

### 2.3. Preparation of Aqueous and Organic Mixtures

The aqueous mixture was prepared from CdSO_4_·H_2_O and CuSO_4_·5H_2_O with 200 mM Na_2_SO_4_ as inert salt in deionized water. Various concentrations of EDTA (10 mM to 100 mM) were added to the aqueous solution, as a masking agent. The organic mixture was prepared with Aliquat 336 as extractant, TBP or TOPO as phase modifier, and toluene as diluent. Different concentrations of Aliquat 336 (50–200 mM) and phase modifier (0–100 mM) were tested. TBP and TOPO were used as phase modifier to reduce the formation of emulsion and enhancement of phase separation during LLE. Karl-Fischer determination for fresh Aliquat 336 had been identified to have 1.7 wt% water, and toluene had 0.03 wt% water. Due to a small amount of Aliquat 336 added into toluene (as diluent) utilized in this experiment, the water content in the mixture of Aliquat 336 in toluene was not significant.

### 2.4. Selective Liquid-Liquid Extraction of Cd(II) and Cu(II) Ions

Extraction studies were conducted by combining 20 mL of the organic mixture (Aliquat 336 and TBP) with the aqueous mixture (100 mg/L Cd(II) and Cu(II) ions) at a ratio of 1:1. The mixture was stirred with an overhead stirrer at 150 rpm for 10 min and left to separate for 5 min. The pH_eq_ of the aqueous mixture was recorded before adjusting to preferable pH by adding in small drops of H_2_SO_4_ or NaOH. The mixture was mixed again and left to separate for 5 min until the desired pH_eq_ was obtained. To collect the separated aqueous sample, an aqueous phase containing extracted metals was obtained after phase disengagement using a separating funnel. The separated aqueous samples were analyzed to determine the concentrations of metal contents, *M*_aq_ of Cu(II) and Cd(II), respectively, with FAAS after appropriate dilution.

Standard error was found to be less than 1% after triplicate runs of the experiment. The percentage of extraction (E%) of metal ions was calculated using Equation (1).

(1) $$\mathrm{Extraction},\;E\;(\%)\;=\;\frac{\left[M_{i}\right]-{[M}_{\mathrm{aq}}]}{{[M}_{i}]}\text{ × }100$$

where *M*_i_ (mg/L) is the initial metal ion concentration in aqueous phase, and *M*_aq_ (mg/L) is the final metal ion concentration after LLE studies.

### 2.5. Screening and Optimization of Operating Parameters

In this study, response surface methodology (RSM) was used for analytical optimization of variables that govern the selective extraction of Cd(II) over Cu(II) ions significantly. All experiments were run in triplicate, and the relative standard deviation between replicated samples was less than 2%. Minitab software (Release 17, Minitab Inc., State College, PA, USA) was used to analyze the data involved in RSM. A 2^5–1^ fractional factorial design was applied to screen five important parameters within their specific ranges, namelym equilibrium pH (pH 2–5), Aliquat 336 concentration Aliquat 336 (50–100 mM), TBP concentration TBP (50–100 mM), concentration of EDTA (10–50 mM), and organic to aqueous ratio (O:A) (1:1 to 2:1) for their significance on the response (E%). Other factors, such as mixing time (10 min), operating temperature (28 ± 1 °C), mixing speed (150 rpm), concentration of inert salt Na_2_SO_4_ (200 mM), initial concentration of Cd(II) and Cu(II) ions *M*_i_ (100 mg/L), diluent type (toluene) were fixed at specific values, based on earlier findings. Sixteen experimental runs were conducted for each metal whereby the range for each parameter was determined based on the earlier findings [26,27].

The optimization experiment was studied using central composite design (CCD) on selective extraction of Cd(II) over Cu(II) ions obtained from the screening experiments. Level 0 represents the center value of each parameter, the low (−1) and high (+1) values, the extreme low (−α) values, and the extreme high (α) values for each parameter studied [28]. Parameters that did not have a significant impact on the selective extraction of Cd(II) over Cu(II), were fixed at low values. Other parameters were fixed as described in screening experiments. The optimum operating conditions for maximum Cd(II) separation from Cu(II) using Aliquat 336-TBP in toluene with EDTA were evaluated by Minitab software. Composite desirability (*D*) was used to evaluate the optimum value of significant parameters by identifying the degree of satisfaction.

A second-order response function in Equation (2) was selected to best fit the response data for multiple regression analysis:

(2) $$y\;=\;\beta_{o}+\overset{k}{\underset{i\;=\;1}{\sum }}\beta_{i}x_{i}+\;\overset{k}{\underset{i\;=\;1}{\sum }}\beta_{ii}x_{i}^{2}+\sum \underset{j<i}{\sum }\beta_{ij}x_{i}x_{j}+\varepsilon \;$$

where *y* is the dependent variable, $\beta_{o}$, $\beta_{i}$, $\beta_{ii}$ and $\beta_{ij}$ are the regression coefficients of intercept, linear, quadratic, and interaction variables, respectively, *x_i_* and *x_j_* are the independent variables, and ε is the error for the effects of excluded parameters. By using Minitab software, the coefficients gathered from optimization experiments, were determined by the least square’s method [28] to best fit the regression model.

## 3. Results and Discussion

### 3.1. Effect of pH on the Extraction of Cd(II) and Cu(II) Ions

**Figure 1:**
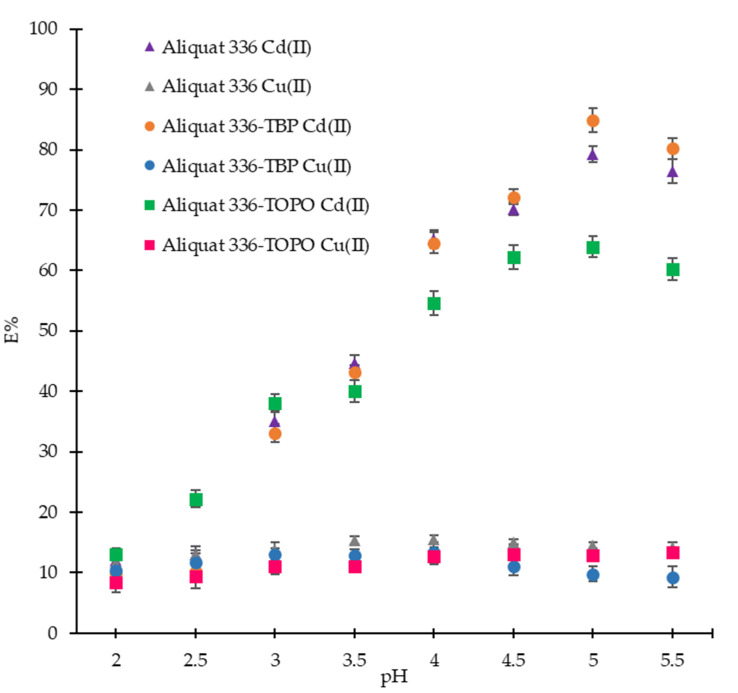
Effect of pH on the extraction of Cd(II) and Cu(II) ions using Aliquat 336 and combinations of Aliquat 336 with TBP and TOPO (mixing time: 10 min; operating temperature: 28 ± 1 °C; mixing speed: 150 rpm; Na_2_SO_4_: 200 mM; diluent: toluene; *M*_i_: 100 mg/L; O:A ratio = 1:1; Aliquat 336: 100 mM; TBP: 50 mM; TOPO: 50 mM).

The operating range of pH in the feed solution determines the rate of transportation of Cd(II) and Cu(II) ions. Extraction had proven to be low at alkaline pH, due to the formation of insoluble metal hydroxide with the appearance of OH^-^ ions in the aqueous mixture and unsuccessful complexation with the extractant [29]. Hence, this study was performed with an aqueous solution with a pH from 2.0 to 5.5. To separate Cd from Cu, experiments were conducted by using a single carrier, Aliquat 336 in toluene, and different combinations of the carrier with phase modifiers, such as Aliquat 336-TBP and Aliquat 336-TOPO, as shown in Figure 1.

The influence of the addition of 50 mM phase modifier (TBP or TOPO) into 100 mM Aliquat 336 on the separation of Cd(II) and Cu(II) ions (initial concentration for both metal ions was 100 mg/L) with the change in pH feed was also examined. Other factors, such as mixing time (10 min), operating temperature (28 ± 1 °C), mixing speed (150 rpm), concentration of sodium sulfate Na_2_SO_4_ (200 mM), and diluent type (toluene) were fixed. The results indicated that the addition of TOPO into Aliquat 336 did not improve the extraction efficiency as compared with Aliquat 336 as a single extractant whereas, Aliquat 336 with TBP in toluene intensified the extraction of Cd(II) over Cu(II) ions at pH 4.5 and more. 100 mM Aliquat 336 and 50 mM TBP enhanced the extraction by having the maximum extraction of Cd(II) (84.93%) with the least extracted Cu(II) (9.79%) at pH 5. In previous literature, Cd(II) transport efficiency was 82% when only 100 mM Aliquat 336 was used as the sole extractant [26]. Combinations of extractants with TBP had also been proven to improve separation efficiency and phase separation [16,30].

### 3.2. Effect of Carrier Concentration

**Figure 2:**
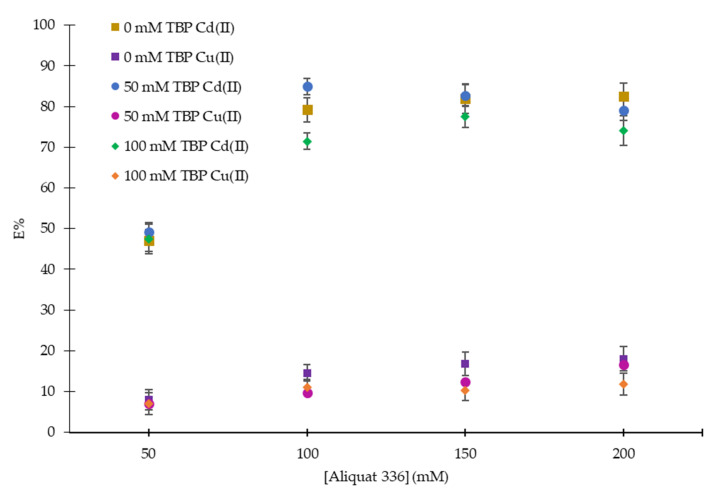
Effect of Aliquat 336 concentration on the extraction of Cd(II) and Cu(II) ions (mixing time: 10 min; operating temperature: 28 ± 1 °C; mixing speed: 150 rpm; Na_2_SO_4_: 200 mM; diluent: toluene; *M*_i_: 100 mg/L; pH_eq_: 5; O:A ratio = 1:1).

The effect of concentration of extractant (Aliquat 336) on the separation of Cd(II) and Cu(II) had also been studied. Concentrations of Aliquat 336 in the range of 50 mM to 200 mM were investigated. The obtained results are shown in Figure 2. As observed when 100 mM of Aliquat 336 in toluene was used, the extraction of Cd(II) ions is almost two times more effective than when 50 mM was used. It may be explained by considering the increasing availability and formation of Cd(II)-Aliquat 336 complex. For extraction of Cd(II) with Aliquat 336, Cd(II) extended its coordination numbers and tended to connect to Cl attached on Aliquat 336. Extraction equilibrium of Cd(II) from sulfate mixture by R_4_N–Cl can be expressed in the Equation (3). Thus, (R_4_N-Cl)_2_·CdSO_4_ is the main extracted complex.

(3) $${\mathrm{Cd}}^{2+}{\;+\;H}_{2}{\mathrm{SO}}_{4}{\;+\;2R}_{4}N\text{-}\mathrm{Cl}\;↔{\;CdSO}_{4}\cdot {\;2R}_{4}{N\text{-}\mathrm{Cl}\;+\;2H}^{+}\;$$

This also shows that Aliquat 336 dissolves satisfactorily in toluene. Results also showed that a further increase in Aliquat 336 concentration after 100 mM did not improve the extraction much. Excess carrier concentration (150 mM to 200 mM) eventually increased the viscosity of the membrane phase, resulting in the reduced mobility and lowered diffusion rate of the metal-carrier complex [28].

Results obtained from Figure 1 and Figure 2 confirmed that the combination of Aliquat 336 with 50 mM TBP is more efficient in the separation of Cd(II) over Cu(II) ions. Without TBP, the extraction of Cu(II) was observed to increase from 8% to 18% as the concentration of Aliquat 336 was increased from 50 mM to 200 mM. However, a further increase in the concentration of TBP up to 100 mM reduced the extraction of Cd(II) as the viscosity of the organic phase increased.

### 3.3. Effect of EDTA as a Masking Agent

**Figure 3:**
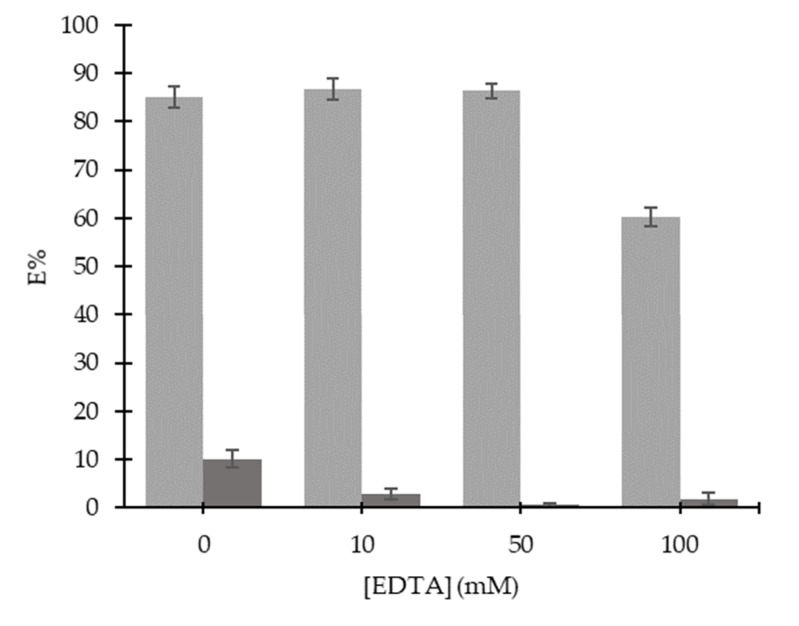
Effect of ethylenediaminetetraacetic acid (EDTA) on the extraction of Cd(II) and Cu(II) ions (Aliquat 336: 100 mM; TBP: 50 mM; diluent: toluene; mixing time: 10 min; operating temperature: 28 ± 1°C; mixing speed: 150 rpm; *M*_i_: 100 mg/L; pH_eq_: 5; O:A ratio = 1:1).

At pH 5, 100 mM Aliquat 336 and 50 mM TBP had proven to extract ~85% of Cd(II) with the least extracted Cu(II). However, ~10% of Cu(II) were co-transported along with Cd(II), and it was difficult to separate Cu(II) from Cd(II) completely. To enhance the separation of Cd(II) over Cu(II), masking agents were introduced to suppress the interference of a particular metal from involving in the reaction without the need of another separation. In current work, EDTA was used as a masking agent to suppress Cu(II) from forming extractable complexes and to improve the selectivity of Cd(II) ions. Concentrations of 10 to 100 mM of EDTA were used to investigate the masking efficiencies of EDTA towards the transport of Cd(II) and Cu(II). Results in Figure 3 showed that the increased EDTA concentration reduced the co-extraction of Cu(II) without reducing the transport efficiency of Cd(II). 50 mM EDTA attained the highest extraction of Cd(II) (76.43%) with Cu(II) almost negligible (0.59%). Cu(II) ions were proven to be masked by 50 mM EDTA in the extraction studies using 100 mM Aliquat 336 with 50 mM TBP at pH 5. However, the extraction of Cd(II) decreased by 26% when 100 mM EDTA was used. The solubility of EDTA was reduced when its concentration increased by more than 50 mM. It can be suggested that EDTA concentration affects the separation of Cd(II) and Cu(II) through the solubility of the masking agent in the medium and the mobility of the metal-complex in the membrane liquid.

### 3.4. Screening of Parameters Affecting the Extraction of Cd(II) and Cu(II) Metal Ions Using 2^5−1^ Fractional Factorial Design

**Figure 4:**
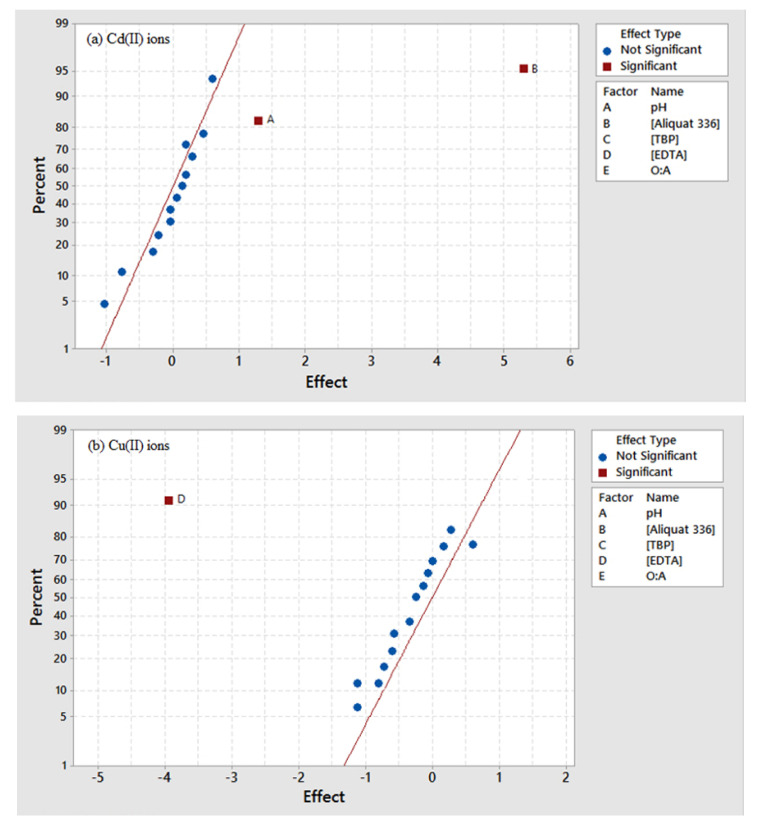
Normal probability plots for (**a**) extraction of Cd(II) ions and (**b**) extraction of Cu(II) ions.

A total of 16 runs in the design matrix for 2^5−1^ fractional factorial design as in Table 2, along with the average E%, were measured in triplicate to screen the factors affecting the extraction of Cd(II) and Cu(II) using Aliquat 336 (extractant), TBP (phase modifier) and EDTA (masking agent). The experimental runs (Std Order) were randomized to reduce the effects of uncontrollable factors. The extraction of Cd(II) was found to range from 8.97 to 89.25%. These factors were evaluated using a probability plot (Figure 4a) at a 5% significance level. Based on the analysis in Figure 4a, significant effects that emerge from the normal probability plot are the main effects of A (pH) and B (Aliquat 336). Concentration of Aliquat 336 was found to influence the extraction of Cd(II) more significantly than the aqueous feed pH. An increase in the concentration of Aliquat 336 resulted in an increase in Cd(II) transport rate, due to a higher amount of free Aliquat 336. At 50 mM to 100 mM of Aliquat 336, the feed/membrane interface was not fully saturated, indicating that the rise in the concentration of extractant could increase the Cd(II) transport efficiency. Aqueous feed pH in this study plays a minor role in the extraction of Cd(II). However, Nayl [31] had proven that separation of divalent metals by increasing the aqueous feed pH by different forms of Aliquat 336. Thus, the best pH for selective extraction of Cd(II) over Cu(II) is fixed at pH 5.

Table 2 also showed the factors affecting the extraction of Cu(II), whereby 16 experimental runs were randomized, and the average E% of Cu(II) was found at 1.08% to 12.02% using the same experimental design for extraction of Cd(II). Figure 4b showed the main effects of the factors that affect E% of Cu(II), and their interactions were also examined using a normal probability plot. It was found that D (EDTA) is the only influential parameter affecting Cu(II) extraction. However, the increase in the concentration of EDTA lowered the Cu(II) transport efficiency significantly. Hence, this could be attributed to the masking effect of EDTA towards the extraction of Cu(II) using Aliquat 336 with TBP.

### 3.5. Optimization of Cd(II) Ions Extraction Using Aliquat 336-TBP in Toluene

#### 3.5.1. Optimization Using Central Composite Design (CCD)

It is noteworthy that two factors, Aliquat 336 and EDTA, have proven to influence the selective extraction of Cd(II) over Cu(II) significantly. Optimization using CCD where each factor was studied in five levels, as shown in Table 3. The factors ranged from 39.65 to 110.35 mM for Aliquat 336 and from 1.72 to 58.28 mM for EDTA. Other controlled factors, such as aqueous feed pH (pH 5), diluent type (toluene), concentration of Cd and Cu in feed phase (100 mg/L), mixing speed (150 rpm), temperature (28 ± 1 °C), mixing time (10mins), O:A ratio (1:1), and TBP (50 mM), were fixed.

Based on 13 experimental runs, the data were used to develop a regression model that correlates E% (response) with the factors, and their adequacies were determined. The best-operating conditions of factors for maximum extraction of Cd(II) were determined, continued by evaluation of their degree of satisfaction. Table 4 shows the design matrix and results of CCD with two factors (D2EHPA (B) and EDTA (D) and one response (extraction%) to extract Cd. The extraction of Cd(II) was found to range from 60.06 to 96.19%.

#### 3.5.2. Regression Model and Analysis of Variance for Cd(II) Extraction

Regression analysis (Table 5) for Cd(II) extraction was performed to find the estimated regression coefficients, along with the corresponding standard deviation (SD_coef_), t-statistics (t-stat), and *p*-values, for linear, square, and interaction terms of a regression model. The calculate probability, *p*-values were less than 0.05, indicated all variable terms were statistically significant. Hence, a second-order polynomial model in coded unit that correlates extraction of Cd(II) is described by Equation (4):

E% = 37.994 + 2.3481 B + 1.3097 D − 0.012152 B*B − 0.015325 D*D − 0.001230 B*D(4)

where B and D are Aliquat 336 and EDTA, respectively. The extent of the regression coefficient resembles the degree of significance of each variable term in the model [32]. From Equation (4), the main effects B and D, showed their synergistic response on the extraction of Cd(II). On the other hand, BD interaction term and quadratic B^2^ and D^2^, showed antagonism on Cd(II) extraction. This shows that an increase in Aliquat 336 and EDTA will boost the separation of Cd(II) over Cu(II), while their squares and interaction terms will eventually reduce Cd(II) extraction.

The adequacy of the regression model for Cd(II) extraction was examined using analysis of variance (ANOVA). The *p*-values from the regression model, as in Table 6, were low (<0.05), whereas the linear, square, and interaction terms indicated that they were statistically significant. Prediction on Cd(II) extraction% was satisfactory by having a high *p*-value of 0.125 (>0.05), based on the lack-of-fit of the model. R^2^ value that is 0.998 (close to 1), implied that the second-order regression model for Cd(II) extraction was satisfactory.

#### 3.5.3. Optimum Conditions for the Extraction of Cd(II) Ions

**Figure 5:**
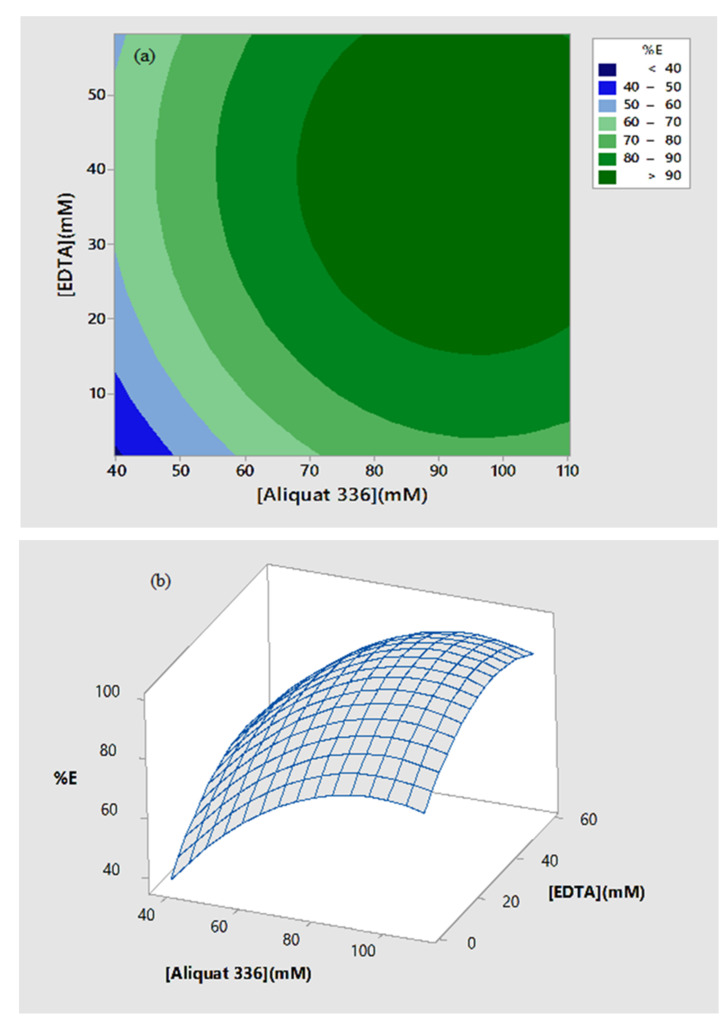
Response surface optimization plots of the extraction of Cd(II) ions against Aliquat 336 and EDTA in (**a**) two-dimensional contour plot and (**b**) three-dimensional response surface plot.

Figure 5a showed a 2D response contour plot of Cd(II) extraction versus Aliquat 336 and EDTA in coded unit. It revealed an increase of % extraction with Aliquat 336 and EDTA, and over 90% of Cd(II) extraction was achieved in the region of 70 to 100 mM of Aliquat 336 and 20 to 50 mM EDTA. The corresponding three-dimensional response surface plot in Figure 5b also reveals similar conditions for the maximum extraction of Cd(II).

**Figure 6:**
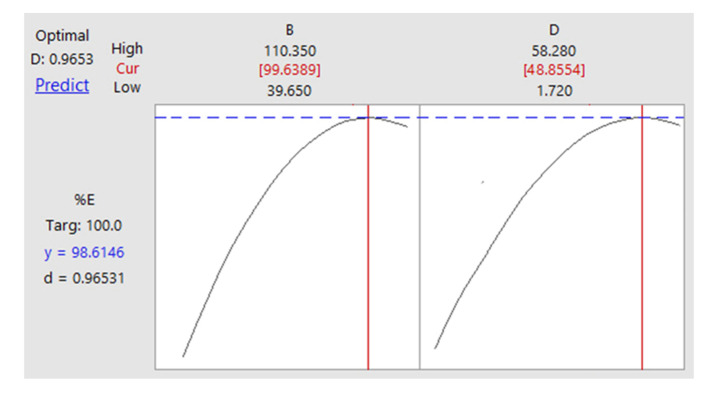
Optimization plot for the extraction of Cd(II) ions; B: Aliquat 336 (mM); D: EDTA (mM).

The optimum conditions of factors in uncoded units (Aliquat 336 = 99.64 mM; EDTA = 48.86 mM) obtained from the optimization plot of % extraction of Cd(II) (Figure 6) generated by the Minitab software with high composite desirability (0.96531).

To maximize the extraction of Cd(II) from the mixture of Cd(II)-Cu(II) (100 mg/L) synthetic solution, experimental runs were conducted under optimum conditions, whereby, 10 min of mixing time, 99.6 mM Aliquat 336, 50 mM TBP, 48.86 mM EDTA, O:A = 1 and 150 rpm of mixing speed using toluene as diluent. The composite desirability of 0.96531 (~1.00) was obtained from Minitab software as listed in Table 7, with a small deviation (2.76%) of the experimental (95.89%) from the predicted (98.61%) Cd(II) extraction % revealed that the regression model in Equation (4) was satisfied.

### 3.6. FTIR Studies

**Figure 7:**
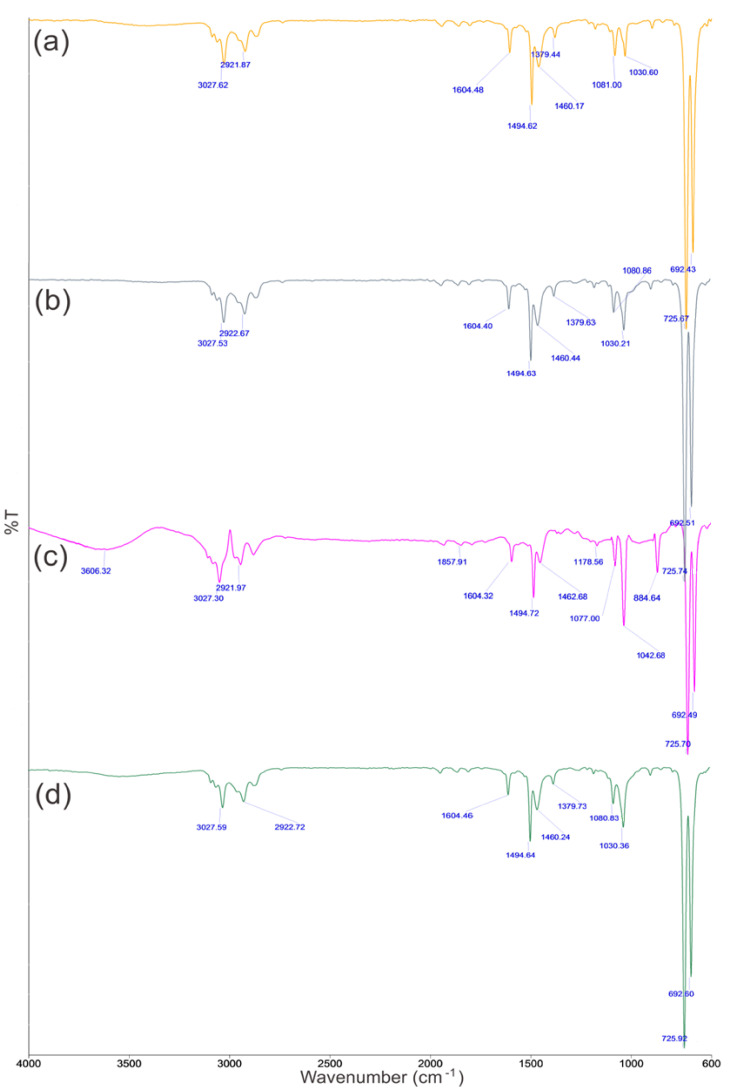
FTIR spectra for (**a**) Aliquat 336 in toluene, (**b**) Aliquat 336-TBP in toluene, (**c**) Cd(II) ions loaded Aliquat 336 and (**d**) spent Aliquat 336 after stripping.

In this study, FTIR spectroscopy was used to identify the interaction between Aliquat 336 and Cd(II) ions. The infrared spectra of the organic phases with single Aliquat 336 (Figure 7a and the mixtures of Aliquat 336-TBP systems in the absence (Figure 7 and the presence of metals (Figure 7) were recorded. The spectrum of spent Aliquat 336 after stripping was also compared in Figure 7d.

From Figure 7, the IR spectrum of the Aliquat 336 was almost identical to the spectrum of Aliquat 336-TBP. It confirmed the role of TBP as a phase modifier rather than playing a role as an extractant for Cd(II) ion. By adding TBP, the efficiency of selective extraction increased by causing the separation of Cd(II) and Cu(II) isotherm. Consequently, Aliquat 336 plays the main role in Cd extraction. The –C–N stretching vibration peak at 1031 cm^−1^ for Aliquat 336-TBP was shifted to 1043 cm^−1^ for Cd(II)-Aliquat 336 complex showing that the bonding between Cd(II) and Aliquat 336 occurred. Stretching of vibration of –O–H of soluble water in Aliquat 336-TBP after extraction of Cd(II) caused the occurrence of a wide adsorption band at 3606 cm^−1^ for Cd(II)-bonded Aliquat 336-TBP [32]. All four spectra showed the characteristic peak for quaternary amine at the peak of 1460 to 1463 cm^−1^, were caused by (CH_3_)N^+^. The asymmetric –C–H peaks (2922 and 3028 cm^−1^) for a mixture of Aliquat 336-TBP before and after extraction studies were similar. The spectrum of spent Aliquat 336 after stripping was almost similar to the fresh Aliquat 336, which confirms the complete stripping of Cd(II) from the loaded Aliquat 336. A similar finding, by Mishra, et al. [33], also verified the reusability of metal loaded Aliquat 336 after the stripping process.

## 4. Conclusions

Investigation on the addition of phase modifier at different pH clearly revealed that TBP enhanced the separability of Cd(II) over Cu(II) at pH 4.5 to pH 5.5. The obtained experimental result had proven the possibility of using Aliquat 336 with TBP to selectively separate Cd(II) with high extractability up to 86.37% over Cu(II) (0.59%) after adding EDTA (50 mM) as a masking agent. Screening of five parameters using a 2^5−1^ fractional factorial design, consisted of equilibrium pH (pH_eq_), Aliquat 336, TBP, EDTA, and organic to aqueous ratio (O:A) revealed that only Aliquat 336 and EDTA have significantly influenced the selective extraction of Cd(II) over Cu(II), followed by optimization with central composite design. A second-order quadratic model was evaluated, and its R^2^ value (0.998) had proven that the model was highly significant and suitable to predict the extraction of Cd(II) in the range of variables studied with a deviation error of 2.76%. The optimum operating parameters for maximum separation of Cd(II) over Cu(II) were determined as follows: Aliquat 336 of 99.64 mM, TBP of 50 mM, EDTA of 48.86 mM, O:A of 1, pH_eq_ of 5, mixing speed of 150 rpm, the operating temperature of 28 °C and mixing time of 10 min. FTIR results have proven that the presence of the interaction between Cd(II) with Aliquat 336. The reusability of Aliquat 336 and its role as the main extractant for extraction of Cd(II) has been proven.

## Figures and Tables

**Table 1:** Standard conditions for Cd(II) and Cu(II) analysis.

Conditions	Cd(II)	Cu(II)
Wavelength (nm)	228.8	324.8
Background correction with D_2_ lamp (1% absorbance in ppm)	0.007–0.015	0.02–0.04
C_2_H_2_ flow rate (L/min)	1.8	1.8
LOD (ppm)	0.002–0.008	0.006–0.02
LOB (ppm)	0.0005–0.0007	0.0014–0.0019
LOQ (ppm)	0.007–0.03	0.02–0.08

**Table 2:** Screening using 2^5−1^ fractional factorial design and average E% of Cd(II) and Cu(II) using Aliquat 336 and TBP in toluene and EDTA as masking agent; diluent: toluene; operating temperature: 28 ± 1°C; mixing speed: 150 rpm; Cd: 100 mg/L; Cu: 100 mg/L.

Std Order	Run Order	pH	Aliquat 336	TBP	EDT	O:A	Average E%	
							Cd(II)	Cu(II)
5	1	2	50	100	10	1:01	9.53 ± 0.43	10.97 ± 0.77
8	2	5	100	100	10	1:01	69.04 ± 1.78	6.11 ± 0.35
3	3	2	100	50	10	1:01	14.49 ± 1.09	11.02 ± 0.92
6	4	5	50	100	10	2:01	56.77 ± 1.97	6.26 ± 0.33
16	5	5	100	100	50	2:01	78.53 ± 1.62	1.73 ± 0.21
10	6	5	50	50	50	2:01	70.12 ± 1.54	1.65 ± 0.43
9	7	2	50	50	50	1:01	8.97 ± 0.94	9.46 ± 0.65
14	8	5	50	100	50	1:01	29.73 ± 1.12	4.61 ± 0.58
13	9	2	50	100	50	2:01	10.66 ± 0.93	8.34 ± 0.49
2	10	5	50	50	10	1:01	52.05 ± 1.84	7.32 ± 0.31
1	11	2	50	50	10	2:01	9.19 ± 0.71	10.76 ± 0.81
7	12	2	100	100	10	2:01	10.48 ± 0.89	12.02 ± 0.95
15	13	2	100	100	50	1:01	12.09 ± 0.52	5.58 ± 0.22
4	14	5	100	50	10	2:01	89.25 ± 1.91	7.44 ± 0.36
11	15	2	100	50	50	2:01	20.98 ± 0.71	7.93 ± 0.51
12	16	5	100	50	50	1:01	84.93 ± 1.12	1.08 ± 0.18

**Table 3:** Five different levels of parameters examined for optimization using CCD.

Parameters	Symbols	Units	Levels				
			−α	−1	0	+1	+α
Aliquat 336	B	mM	39.65	50	75	100	110.35
EDTA	D	mM	1.72	10	30	50	58.28

**Table 4:** Central composite design for optimization of Cd(II) ions extraction (diluent: toluene; mixing time: 10 min; operating temperature: 28 ± 1 °C; TBP: 50 mM; mixing speed: 150 rpm; *M*_i_: 100 mg/L; pH_eq_: 5; O:A ratio = 1:1).

Run	Factors *		Extraction%
	B	D	
1	50.00	50.00	72.92 ± 1.03
2	110.35	30.00	94.47 ± 1.98
3	75.00	30.00	92.52 ± 1.83
4	75.00	30.00	92.48 ± 1.94
5	75.00	30.00	92.51 ± 1.14
6	75.00	30.00	92.44 ± 2.09
7	50.00	10.00	60.18 ± 1.22
8	75.00	58.28	88.91 ± 0.92
9	75.00	1.72	71.48 ± 1.05
10	100.00	10.00	85.91 ± 1.66
11	100.00	50.00	96.19 ± 1.73
12	39.65	30.00	60.06 ± 1.81
13	75.00	30.00	92.49 ± 1.98

* B: Aliquat 336 (mM); D: EDTA (mM).

**Table 5:** Estimated regression coefficients of extraction of Cd(II) using Minitab software; B: Aliquat 336 (mM); D: EDTA (mM).

Term	Coefficient	SD_coef_	t-Stat	*p*-Value
Constant	37.994	0.953	39.88	<10^−4^
B	2.3481	0.0220	106.60	<10^−4^
D	1.3097	0.0218	60.14	<10^−4^
B*B	−0.012152	0.000138	−88.08	<10^−4^
D*D	−0.015325	0.000216	−71.09	<10^−4^
B*D	−0.001230	0.000227	−5.41	<10^−4^

**Table 6:** ANOVA of regression model for extraction of Cd(II); B: Aliquat 366 (mM); D: EDTA (mM); DF: degrees of freedom; SS: sum of squares; MS: mean square.

Source	DF	SS	MS	*F*-Value	*p*-Value
Regression	5	2065.85	413.171	7991.15	<10^−4^
B (Linear)	1	587.58	587.581	11364.41	<10^−4^
D (Linear)	1	186.98	186.983	3616.45	<10^−4^
B*B (Square)	1	401.13	401.133	7758.32	<10^−4^
D*D (Square)	1	261.27	261.275	5053.32	<10^−4^
B*D (Interaction)	1	1.51	1.513	29.26	<10^−4^
Residual error	7	0.66	0.052		
Lack-of-fit	3	0.36	0,119	1.94	0.125
Pure error	4	0.30	0.001		
Total	12	2066.52			
SD_reg_	0.353				
R-squared	0.998				

**Table 7:** Model validation with optimum operating conditions for the extraction of Cd(II).

Composite Desirability	Aliquat 336 (mM)	EDTA (mM)	Predicted E%	Experimental E%	% Deviation
0.96531	99.64	48.86	98.61	95.89	2.76
